# Calcinosis cutis der Unterschenkel – Hyperphosphatämische familiäre tumorale Kalzinose bei einem Patienten mit *GALNT3*‐Mutation

**DOI:** 10.1111/ddg.15716_g

**Published:** 2025-09-15

**Authors:** David Ranzinger, Tobias Schäfer, Franziska Schauer, Kilian Eyerich, Anna Caroline Pilz

**Affiliations:** ^1^ Klinik für Dermatologie und Venerologie Universitätsklinikum Freiburg; ^2^ Klinik für Innere Medizin Universitätsklinikum Freiburg

Sehr geehrte Herausgeber,

Wir berichten über einen 39‐jährigen Patienten europäischer Abstammung, der sich in unserer Ambulanz mit subkutanen Indurationen an beiden Unterschenkeln vorstellte. Diese befanden sich vornehmlich ventral und medial, wurden erstmals etwa 3 Jahre zuvor bemerkt und verursachten ziehende Schmerzen bei Druckausübung (Abbildung 1). In der Anamnese gab der Patient eine linksseitige Nierenagenesie und eine arterielle Hypertonie an, weitere Erkrankungen oder Hautkrankheiten in der Familienanamnese wurden verneint.

**ABBILDUNG 1:**
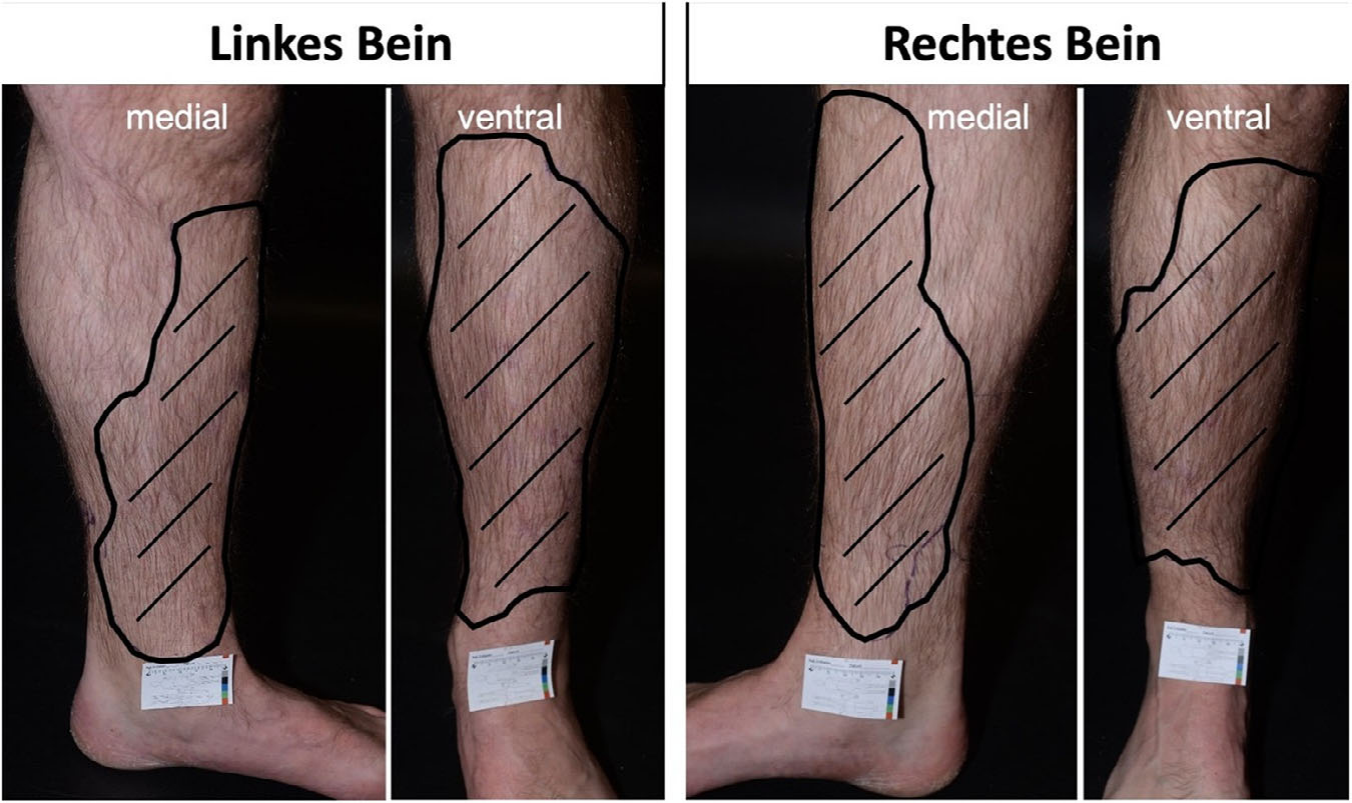
Ausmaß der subkutanen Verkalkungen des linken und rechten Unterschenkels, dargestellt durch den eingekreisten, gestrichelten Bereich.

In der histologischen Untersuchung präsentierten sich amorphe Kalziumpräzipitate am Übergang von der Dermis zur Subkutis und innerhalb der Subkutis ohne Zeichen einer Entzündung.

Die Ursachen der Calcinosis cutis und somit auch ihre Differenzialdiagnosen sind vielfältig. Dystrophische Varianten werden zum Beispiel durch erbliche und/oder autoimmune Bindegewebserkrankungen wie systemische Sklerose oder Dermatomyositis ausgelöst und die metastatische Calcinosis cutis entsteht durch einen abnormen Kalzium‐ und/oder Phosphatstoffwechsel.

Laboruntersuchungen zeigten erhöhte Phosphatwerte mit 1,8 mmol/l (0,81–1,45 mmol/l) und normale Kalziumwerte mit 2,49 mmol/l (2,09–2,56 mmol/l). Die Parathormonwerte lagen mit 22 pg/ml (15–65 pg/ml) im unteren Normbereich. Die Nierenfunktion war normal und es wurde keine signifikante Proteinurie festgestellt. Die Beurteilung der tubulären Funktion ergab eine tubuläre Rückresorption von Phosphat von nahezu 100 % (80 %–95 %). Folglich wurde eine hyperphosphatämische familiäre tumorale Kalzinose (HFTK) vermutet. Um dies zu bestätigen wurde eine vollständige Exom‐Sequenzierung von peripherem Blut durchgeführt, wobei eine homozygote Mutation im Gen NM_004482.4 (*GALNT3*) identifiziert wurde.

Die HFTK ist eine außerordentlich seltene Krankheit, die vor allem Menschen aus dem Nahen Osten oder afroamerikanischer Abstammung betrifft^1^ und durch eine Resistenz gegen oder einen Mangel an *fibroblast growth factor 23* (FGF23) verursacht wird. Dieses Hormon reguliert den Phosphatspiegel im Körper.^2^

Störungen, an denen FGF23 beteiligt ist, führen aufgrund erhöhter renaler tubulärer Phosphorrückresorption zu einer Hyperphosphatämie, während der Kalziumspiegel im Blut und die Nierenfunktion unbeeinträchtigt bleiben.^3^

Die zugrundeliegenden Mutationen hierfür finden sich im *FGF23*‐Gen, im *KL*‐Gen, das für Klotho, einen wichtigen Co‐Rezeptor für die FGF23‐Signalübertragung, kodiert und am häufigsten im *GALNT3*‐Gen. *GALNT3* kodiert für die N‐Acetylgalactosaminyltransferase 3, ein Enzym, das die O‐Glykosylierung von Proteinen katalysiert und eine entscheidende Rolle bei der Kontrolle der FGF23‐Aktivität spielt.^4^

Im Genom unseres Patienten wurde eine homozygote c.516‐2A>G‐Mutation im *GALNT3*‐Gen identifiziert. Die Transition von Adenin zu Guanin befindet sich in der Akzeptor‐Spleißstelle von Intron 1. Bislang wurde diese Mutation in der Literatur nur einmal von Masi et al. beschrieben.^5^ Mit Hilfe eines computergestützten Vorhersageinstruments prognostizierten Masi et al., dass die Mutation zu einem Überspringen von Exon 2 während des Spleißvorgangs führt (Abbildung 2).^5^

**ABBILDUNG 2:**
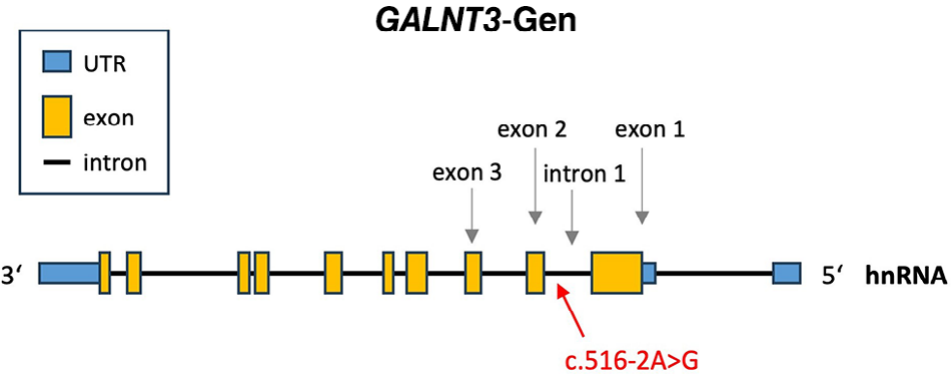
Transition von Adenin zu Guanin in der Akzeptor‐Spleißstelle von Intron 1.

Andere Autoren haben bereits einen Adenin‐Thymin‐Austausch an derselben Stelle beschrieben, wobei Laleye et al. experimentell nachweisen konnten, dass die A>T‐Mutation tatsächlich zu einem Überspringen von Exon 2 und einer Leserasterverschiebung führt.^2^, ^6^, ^7^ Infolge dessen entsteht ein vorzeitiges Stopp‐Codon nach vier Aminosäuren im Exon 3, was zu einem verkürzten, wahrscheinlich nicht funktionalen Protein führt.^7^ Ein ähnliches Ergebnis kann für die A>G Transition erwartet werden.

Das klinische Erscheinungsbild der HFTK ist sehr variabel, allerdings treten tumorale Verkalkungen typischerweise in Bereichen, die wiederholten Traumata oder Druck ausgesetzt sind wie Ellbogen, Schultern, Hüften und Knie sowie in den ersten beiden Lebensjahrzehnten auf.^2^ Verkalkungen können auch extrakutanes Gewebe betreffen, hierzu zählen Hoden, Dura, Nieren, Augenlider, Netzhaut (angioide Streifen) und Blutgefäße (vaskuläre Verkalkungen).^8^, ^9^ Außerdem wurde über Hyperostosen der Diaphysen sowie Zahnveränderungen (zum Beispiel Wurzelverkürzung, Pulpaobliteration) berichtet.^2^

Bei unserem Patienten wurden bei einer multiregionalen CT‐Untersuchung Weichteilverkalkungen subgaleal, dorsal der Olekranone und an den Fußrücken sowie arteriosklerotische Veränderungen an der Aorta, den Beckengefäßen, den Unterarm‐ und den Unterschenkelgefäßen festgestellt (Abbildung 3). Es konnten korneale Kalkablagerungen, aber keine dentalen Veränderungen diagnostiziert werden.

**ABBILDUNG 3:**
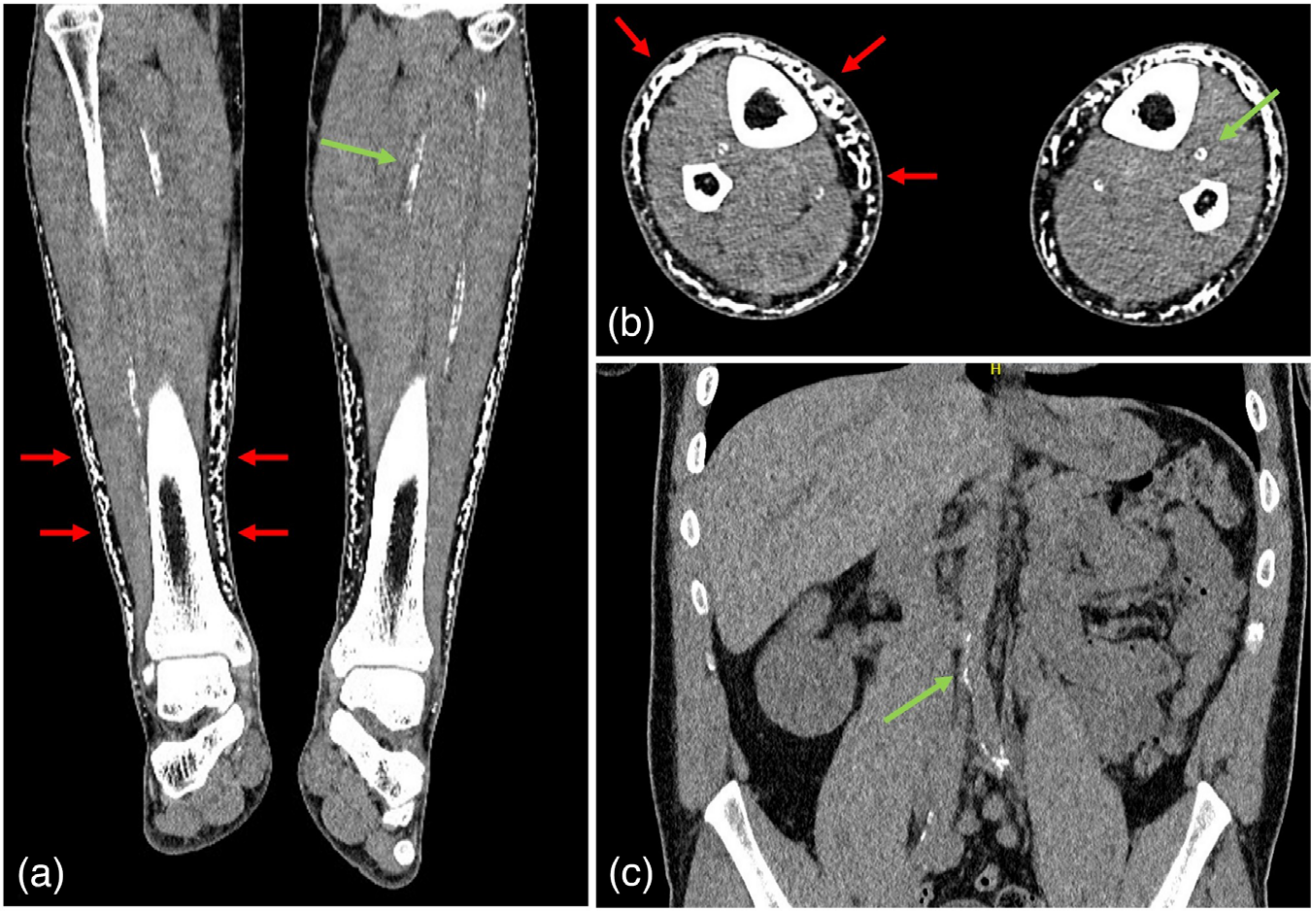
Unterschenkel (a) im Frontal‐ und (b) Querschnitt. (c) Abdomen im Frontalschnitt. Subkutane Verkalkungen an den Unterschenkeln, die teilweise den gesamten Umfang betreffen (rote Pfeile). Die arteriellen Gefäße der Unterschenkel und die Aorta abdominalis mit den absteigenden Iliakalgefäßen zeigen ausgeprägte Verkalkungen (grüne Pfeile).

Unser Patient wurde zunächst mit einer phosphatarmen Diät und 2,4 g des Phosphatbinders Sevelamercarbonat täglich behandelt. Da die Phosphatwerte nach 6 Monaten nicht sanken, wurde die Dosis auf 4,8 g pro Tag erhöht. Die arterielle Hypertonie wurde mit Ramipril behandelt und bezüglich der ophthalmologischen Beteiligung war keine Intervention erforderlich. Da die Folgetermine in unserer Klinik durch den Patienten nicht wahrgenommen wurden, konnte der weitere Krankheitsverlauf nicht nachverfolgt werden.

In diesem Bericht wird der zweite Fall einer HFTK mit einer c.516‐2A>G‐Mutation im *GALNT3*‐Gen vorgestellt. Im Gegensatz zur Mehrheit aller beschriebenen HFTK‐Fälle sind unser Patient und die von Masi et al. beschriebene Patientin beide europäischer Abstammung. Beide waren hauptsächlich an den unteren Extremitäten betroffen, zeigten intrakranielle und vaskuläre Verkalkungen, aber keiner von ihnen hatte Zahnveränderungen und/oder angioide Streifen. Daher könnte die c.516‐2A>G‐Mutation eine vorwiegend bei Kaukasiern vorkommende Variante darstellen, die zu einem speziellen HFTK‐Phänotyp führen könnte.

## DANKSAGUNG

Open access Veröffentlichung ermöglicht und organisiert durch Projekt DEAL.

## INTERESSENKONFLIKT

Keiner.
